# Somatic production of reactive oxygen species does not predict its production in sperm cells across *Drosophila melanogaster* lines

**DOI:** 10.1186/s13104-021-05550-7

**Published:** 2021-04-07

**Authors:** Biz R. Turnell, Luisa Kumpitsch, Anne-Cécile Ribou, Klaus Reinhardt

**Affiliations:** 1 Applied Zoology, Institute of Zoology, Technische Universität Dresden, Zellescher Weg 20b, Raum 258, 01069 Dresden, Germany; 2 Institut de Modélisation Et D’Analyse en Géo-Environnement Et Santé, Université de Perpignan Via Domitia, 52 Avenue Paul Alduy, 66860 Perpignan, France

**Keywords:** Alternative oxidase, *Dj-1*β, *DJ-1*, Oxygen radicals, Antioxidants, Sperm ageing, Sperm storage

## Abstract

**Objective:**

Sperm ageing has major evolutionary implications but has received comparatively little attention. Ageing in sperm and other cells is driven largely by oxidative damage from reactive oxygen species (ROS) generated by the mitochondria. Rates of organismal ageing differ across species and are theorized to be linked to somatic ROS levels. However, it is unknown whether sperm ageing rates are correlated with organismal ageing rates. Here, we investigate this question by comparing sperm ROS production in four lines of *Drosophila melanogaster* that have previously been shown to differ in somatic mitochondrial ROS production, including two commonly used wild-type lines and two lines with genetic modifications standardly used in ageing research.

**Results:**

Somatic ROS production was previously shown to be lower in wild-type Oregon-R than in wild-type Dahomey flies; decreased by the expression of alternative oxidase (AOX), a protein that shortens the electron transport chain; and increased by a loss-of-function mutation in *dj-1*β, a gene involved in ROS scavenging. Contrary to predictions, we found no differences among these four lines in the rate of sperm ROS production. We discuss the implications of our results, the limitations of our study, and possible directions for future research.

**Supplementary Information:**

The online version contains supplementary material available at 10.1186/s13104-021-05550-7.

## Introduction

Like sexually reproducing organisms, sperm cells have a life cycle during which they develop, age, and die. As in organisms, the ageing process in sperm cells leads to declines in both performance and fitness outcomes. Older sperm cells have reduced motility [1], velocity [2, 3] and fertilization capacity [4], and produce fewer and less viable offspring [4–6]. Sperm ageing has major consequences for sexual selection. On the male side, it may account for some of the wide, largely unexplained variation in competitive fertilization success [7–9]. On the female side, sperm ageing may influence both pre- and post-copulatory behaviour, driving frequent mating to refresh old sperm stores [7] and providing a potential mechanism for cryptic choice [10]. As in somatic cells, ageing in sperm is driven in large part by reactive oxygen species (ROS). When more ROS are produced than are scavenged by antioxidants, they can damage cellular components like lipids, proteins, and DNA. Sperm cells are particularly vulnerable to this oxidative stress, both because of their limited antioxidant reserves and because of the high concentration of oxidation-prone polyunsaturated fatty acids in their membranes [1].

Most cellular ROS are produced by the mitochondria during aerobic respiration. Production rates vary widely across species and are often negatively correlated with lifespan, as predicted by the mitochondrial free radical theory of ageing [11]. ROS production also commonly varies with tissue type [12–14], and cross-species differences in ROS production can themselves be tissue-dependent; that is, production may be higher in one species than in another, but only in certain tissues [15]. These species by tissue interactions raise the question of whether the ageing rate of sperm is at all related to the ageing rate of the organism producing them [16]. Such a relationship would have significant implications for the intersection of life history patterns and sexual selection. To our knowledge, however, no studies have compared sperm ROS production rates within or across species.

Here, we measure rates of sperm ROS production and compare them to previously reported rates of somatic mitochondrial (mt) ROS production in four *Drosophila melanogaster* lines: (1) wild-type Oregon-R; (2) wild-type Dahomey; and two genetically modified lines expressing, in the Dahomey background, either (3) alternative oxidase (AOX), a protein that decreases ROS formation by bypassing part of the electron transport chain; or (4) or a loss-of-function mutation in *dj-1*β, a gene involved in ROS scavenging. Sanz et al. [17], as predicted, found a negative correlation between lifespan and whole-body mtROS production across the two wild-type lines, with Oregon-R flies living longer and producing less ROS than Dahomey flies. In contrast, lifespan did not differ between flies expressing AOX, which produced more mtROS, and mutant *dj-1*β flies, which produced less.

We predicted that sperm ROS production would mirror somatic mtROS production, being lowest in in Oregon-R and in AOX-expressing flies, moderate in Dahomey flies, and highest in mutant *dj-1*β flies.

## Main text

### Materials and methods

#### Fly lines and husbandry

The Oregon-R line (referred to hereafter as “OR”) is a wild-type line widely used in *Drosophila* research. UAS-AOX F6 [18], *daughterless*-Gal4 driver (daGal4) (previously BL55849) [19], *dj-1*β^*GE23381*^ [20], and *white* wild-type Dahomey [21] (wDAH, referred to hereafter as “DAH”) flies were kindly provided by Dr. Alberto Sanz (then University of Newcastle). The UAS-AOX F6, daGal4, and *dj-1*β lines had been backcrossed into the DAH background for 11, 11, and 7 generations, respectively [17]. In the current study, AOX-expressing AOX/daGal4 flies (referred to hereafter as “AOX”) were generated by mating UAS-AOX F6 males to virgin daGal4 females. Flies were maintained at 25 °C and 60% humidity on a 12:12 light:dark cycle and fed on a yeast-corn-sugar medium (40 g/l yeast, 90 g/l corn meal, 100 g/l sucrose, 12 g/l agar, 40 ml/l nipagin [10% in ethanol] and 3 ml/l propionic acid in water). Males were collected upon eclosion and kept in groups of three to eight individuals.

#### Rate of reactive oxygen species production measured by time-resolved microfluorimetry

The rate of ROS production in sperm was measured as described in [14]. Briefly, time-resolved microfluorimetry of the oxygen probe 1-pyrene butyric acid (PBA) was used to measure the in situ production rate of ROS. PBA’s fluorescence lifetime decreases upon collision with small ROS molecules like superoxide, but not upon collision with the stable, accumulating ROS molecules like hydrogen peroxide into which they transform [22]. PBA lifetime is unaffected by probe concentration and cell number [23]. Seminal vesicles from three-to-six-day-old virgin males were dissected in PBS and punctured to release the sperm, which were then incubated in 20 μl of 1 μM PBA in 2% ethanol on a coverslip for four minutes and washed three times with PBS to remove the excess probe. Sperm were laser-excited as described previously to generate the fluorescence decay curve [14]. Eight readings per sample were taken, resulting in eight decay curves that were then averaged to obtain the mean fluorescence lifetime. Relative rates of ROS production were calculated using the Stern–Volmer equation [14, 24]. All statistics were performed in R version 3.6.1 [25].

### Results

**Fig. 1:**
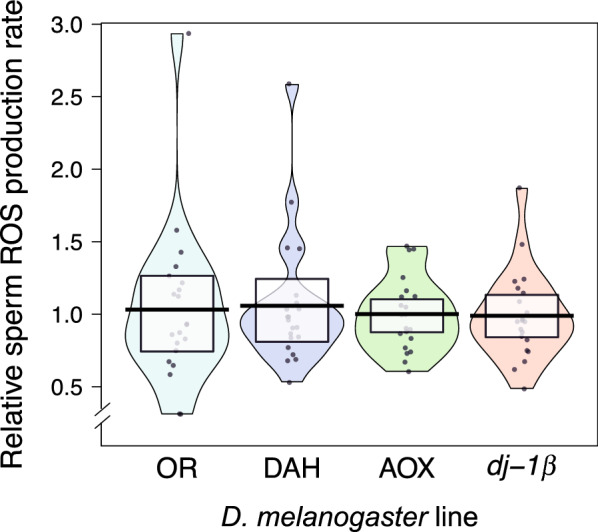
Relative rate of production of reactive oxygen species in sperm from Oregon-R, Dahomey, AOX, and mutant *dj-1*β males, as measured by the fluorescence lifetime of the oxygen probe 1-pyrene butyric acid (PBA) and calculated by the Stern-Vomer equation (see text for details). Black line = mean, white box = 95% CI

Sperm ROS production rates, being non-normally distributed (Shapiro–Wilk normality test, *p* < 1e−7), were compared using a Kruskal–Wallis rank sum test. There was no difference across lines in the rate of ROS production in sperm (Fig. 1; *χ*^2^ = 0.10, *p* = 0.99, *n* = 21 per line; see Additional file 1 for raw data).

### Discussion

Sperm ageing, an underappreciated phenomenon, has the potential to drive selection on traits in males and females, pre- and post-mating, and at the gametic and organismal levels. Because sperm ageing shares a mechanism with somatic ageing—oxidative stress via ROS—a correlation between the rates of sperm and somatic ageing, whether across species or individuals, may be predicted. Here, we find no evidence for such a correlation. Contrary to expectations, sperm from wild-type Oregon-R (OR) flies, which have lower somatic mtROS production and longer lifespans [17], did not produce less ROS than sperm from high-mtROS, shorter-lived wild-type Dahomey (DAH) flies. More surprisingly, sperm ROS production did not differ between DAH and either of the two genetically modified lines with a DAH background: one expressing alternative oxidase (AOX) and producing less somatic mtROS, and one expressing a loss-of-function *dj-1*β mutation and producing more somatic mtROS.

Post-meiotic *D. melanogaster* sperm have been shown to undergo oxidative phosphorylation [14, 26], generating mtROS in the process [14, 27]. Sperm expressing AOX, which bypasses a major site of mtROS formation in the electron transport chain, complex III [28], would therefore be expected to show lower ROS production. Given our results, it seems likely that, while AOX is expressed in the testes and seminal vesicles of AOX males [29], it is not expressed in the germline itself. Although the daGal4 driver is supposedly ubiquitously expressed, it is not unusual for “ubiquitous” drivers to show no expression in germ cells [30]. Indeed, a different AOX construct driven by the also ubiquitous *α-tubulin* promoter was not expressed in the *D. melanogaster* male germline [29].

Unlike the AOX gene, the mutated *dj-1*β gene should, in theory, certainly be expressed in the germline. However, the dj-1*β* protein in *Drosophila* may chiefly promote the scavenging of hydrogen peroxide [31], a non-radical ROS whose production is not detected by our method. It is thus possible that mutant *dj-1*β sperm accumulates a greater amount of stable ROS than DAH sperm despite producing small radical ROS like superoxide at the same rate.

While sperm from the four lines did not differ in their rates of endogenous ROS production, they are likely to experience different levels of exogenous mtROS produced by the testes and seminal vesicles during spermatogenesis and storage. Sperm from AOX males may thus be expected to accumulate age-related cellular damage more slowly, despite producing normal amounts of ROS. Likewise, sperm from mutant *dj-1*β males may age more quickly due to decreased antioxidant production in the surrounding reproductive tissues, which would lead to less scavenging of ROS both exogenous and endogenous to the sperm.

The rate of sperm ageing may also be affected by the post-mating environment of the female reproductive tract. Sperm transferred to females in lines with low somatic mtROS production may survive longer in storage, regardless of their endogenous ROS production. Females may also mitigate the effects of mtROS, whether originating from the stored sperm itself or from the surrounding female tissues, by increasing antioxidant production. Antioxidant genes are upregulated in the sperm storage organs of mated females in *D. melanogaster* [32, 33] (reviewed in [34]), honeybees [35, 36], and ants [37], and high antioxidant levels have also been found in the female sperm storage organs of mammals and birds [38].

The rate of sperm ageing is likely affected by both male and female factors. Indeed, in a related study, we found indications of slower sperm ageing both in AOX sperm stored by control females and in control sperm stored by AOX females, as evidenced by the increased reproductive output of these crosses. Conversely, crosses involving mutant *dj-1*β males or females had decreased reproductive output, consistent with faster ageing in control-stored *dj-1*β sperm and in *dj-1*β-stored control sperm [39].

In conclusion, we predicted that sperm ROS production rates would correlate with somatic mtROS production rates across *D. melanogaster* lines. To our knowledge, this is the first study to compare sperm ROS production within or across species. While we found no between-line differences in the rates of sperm-endogenous ROS production, it is nonetheless possible that the lines differ in their rates of sperm ageing. Measuring cellular damage markers and antioxidant activity in male- and female-stored sperm, as well as the fitness outcomes of the individuals transferring and using that sperm, would help to shed further light on the factors influencing sperm ageing and on its evolutionary consequences.

## Limitations

The fly lines used in this study were obtained directly from the authors of [17]. We did not independently verify either their genotypes or their rates of somatic mtROS production.

## Supplementary Information

**Additional file 1.** Fluorescence lifetime of PBA and relative rates of sperm ROS production.

## Data Availability

All relevant data are included in additiona file.

## Abbreviations

AOX: Alternative oxidase
DAH: Wild-type Dahomey
mtROS: Mitochondrial reactive oxygen species
OR: Wild-type Oregon-R
PBA: 1-Pyrene butyric acid
ROS: Reactive oxygen species
